# The Systems Measurement of Mammalian Biotas, Part Two

**DOI:** 10.3390/life13112193

**Published:** 2023-11-10

**Authors:** Charles H. Smith, Patrick Georges, Ngoc Nguyen

**Affiliations:** 1 Western Kentucky University (Prof. Emeritus), Bowling Green, KY 42101, USA; 2 Graduate School of Public and International Affairs, University of Ottawa, Ottawa, ON K1N 6N5, Canada; pgeorges@uottawa.ca; 3 Department of Mathematics, Western Kentucky University, Bowling Green, KY 42101, USA; ngoc.nguyen@wku.edu

**Keywords:** mammals, faunal classification, natural systems, evolution, distribution patterns, maps

## Abstract

For a recent publication, the authors identified a seven-region model of mammal family distribution patterns, in which each unit contributes equally to the system’s overall statistical characteristics of diversity, despite its individual units having measurably different levels of diversity and endemism. This systemization presents a highly efficient descriptive model that can possibly be interpreted as a form of natural classification. An additional analysis of the same mode is described here, in which the seven-region model of the distribution of mammal families’ spatial affinities is shown to closely approach a most-probable-state arrangement, as assessed through combinatorics, raising some important questions about how macroevolutionary patterns might self-organize spatially. One of the possible practical applications of the overall approach is to areal representation; statistical moments of the underlying world patterns can be used to characterize faunal statuses at any individual location by relating the latter to the former. Through this approach, classical concepts such as corridors, tracks, and transition zones might be re-examined in a manner that better lends itself to hypothesis testing. An arbitrarily chosen bounded area, the conterminous United States, is treated in this fashion by way of illustration.

## 1. Introduction

In a recent study, Smith et al. [1] described a new mammal faunas regionalization scheme based on the data of Holt et al. [2] and an analysis format first employed in 1983 by Smith [3,4] focusing on family-level patterns. These latter papers featured geographic entropy maximization methods [5,6] and a new argument for understanding relative regional equivalencies; as mentioned in [1], they have received favorable comments from Whittaker et al. [7] (p. 2210) and Wilson [8] (p. 868), among others.

The 2023 analysis [1] extended this basically phenetic representation by suggesting the possibility that the results could also be interpreted within a framework of “natural systems” thinking. The result was a seven-region system that followed the methods introduced in [3,4], while also conforming to a concept of natural systems organization derived from the natural philosophy of Spinoza [9,10,11,12,13] (which, among other things, posits the existence of the two fundamental organizing properties of existence that he terms “Attributes”—specifically, the Attributes “Spatial Extension,” and “Thought”, which can be viewed as super-”rules of order” that universally back all individually manifest expressions of natural order).

A third Smith paper in 1983 [14] applied statistical moments derived from the data of the first two to construct cartographic representations of worldwide mammalian faunal relations, for example, spatially illustrating varying mean and total cosmopolitanism statistics (these are statistical summaries of the varying proportions of widely distributed versus more restricted forms from region to region; see below for formal derivations) at a sample of worldwide point locations. The present work represents an extension of [1], featuring an addition to the theoretical arguments presented there, and, in the spirit of [14], further suggestions as to the potential application of this manner of faunal region construction to the practical cartographic representation of distribution patterns.

## 2. Materials and Methods

### 2.1. The Equivalence of Regions Question

Holt et al.’s 2013 study [2], integrating phylogenetic considerations into the spatial clustering algorithm, produced a hierarchical inclusion tree starting with 36 initial mammalian spatial units. 

**Figure 1:**
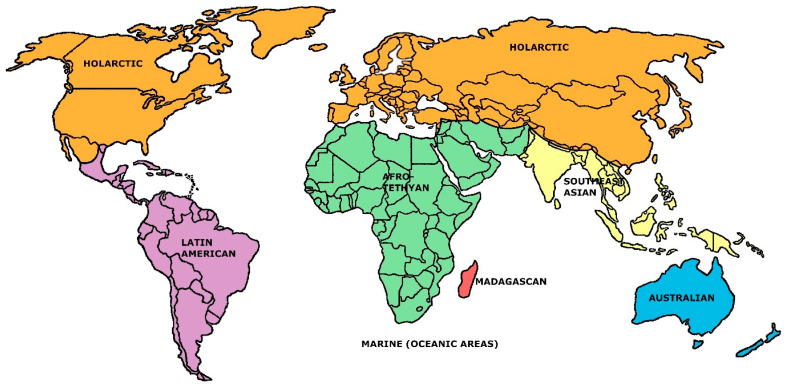
The seven-region, family-based model identified in [1] (applying data presented in Holt et al. [2], (Figure S4C), but adding in an oceanic “Marine Region”).

Figure 1 depicts, in diagrammatic form, the thirtieth level of inclusion in that hierarchy, corresponding to a six-region outcome that is depicted as seven here, as the Holt et al. analysis did not consider marine forms. Holt et al.’s work also did not address the question of what specific number of faunal regions might best portray overall mammalian biogeographic patterns (as in the old Wallace/Sclater system [15,16,17]). This lapse is perhaps understandable as the result of “hierarchy bias”, that is, a sense that a hierarchically organized arrangement of geographical inclusiveness not strictly falling out according to the philosophy of cladistics is “unnatural” to begin with (and thus that there is little profit in trying to identify a single preferred level of regional partitioning). But beyond the cladism assumption, there is a further bias stemming from more than a century’s worth of discussion on the fact that the regions advanced in any given faunal system, however arrived at, are never structurally “equivalent”: some regions will always sponsor a lower or higher percentage of endemic taxa than others, and/or display a lower or higher familial richness [18]. For example, the Palaearctic and Nearctic regions in the Wallace/Sclater scheme have different diversities and/or endemism rates than the same system’s Australasian region. But, and to repeat the question posed in [1], should it really be considered a given that “faunally equivalent” components in this sense are to be expected from the diversification process inherent in a probabilistically evolving, but spatially constrained, biological system?

Smith’s 1983 papers [3,4] exposed the “hierarchy bias” assumption by showing how structural diversity in a bioregionalization sense can be defined in ways extending beyond simple tallies of inclusive forms. These elements of self-specification might be viewed as part and parcel of species line divergences that probabilistically arise in response to “very complex long-term interactions among biological/environmental/geologic constraints and opportunities” [1], (p. 2 of 15).

The seven-region model (Figure 1) developed in [1] is shown to be self-specifying at even considerably higher levels of significance than the model developed in 1983 [3], suggesting a stochastic evolutionary process leading to regional units of greatly varying family richness and number of endemic forms. These are yet “equivalent” to the extent that, organized as they are, they define a system of higher information content. This leads to the suggestion that the seven-region system presented in [1] may not merely be a statistical optimization, but reflective of a natural and recurring pattern of internal differentiation that falls out in a manner of observing certain principles of combinatorial likelihood, and/or negentropy. (Further treatment of the underlying model and its Spinozian foundations may be found in [1,19,20] and online at http://people.wku.edu/charles.smith/once/writings.htm#2 (accessed on 23 June 2023).

### 2.2. A Further “Natural Systems” Argument

The seven-region model of mammal family distribution presented in [1] breaks with the assumption that regional classifications featuring primary units of differing size and diversity need be considered as suboptimal structures; indeed, it is possible to make the argument that such systems may be more likely to evolve stochastically in a manner leading to component units that are not equivalent in that sense. Nevertheless, the data in [1] clearly show that each such component in the system brings with it characteristics that are equally important to specifying conditions across the rest of the system. 

Since the publication of [1], we have completed an investigation of another characteristic of world mammal faunal patterns that tends to support both the seven-region model over the other four entertained, and the “natural law” interpretation. Before switching to a simpler notation for the 7-region model, let us first keep the more general notation that assumes five different models, where the world can be rearranged or decomposed into either 5, 6, 7, 8, or 9 variably sized geographical regions, with the total number of regions included in each model being a variable noted as *r*, with *r* = 5,…9. Each geographical region in any such model will be referred with the index *i* = 1, 2,…, to *r*. Mammal families are indexed as *f* = 1 to *N,* where *N* is the total number of mammal families in the world (140, after [21]), as applied here. Now, let us denote the following:

*d_f,i,r_* for *f* = 1,…, *N*, *i* = 1,…, *r*, *r* = 5,…, 9: a dummy variable that takes a value of 1 if there is such a mammal family (*f*) in region *i* of the *r*-region model, and 0 otherwise. The collection of *d_f,i,r_* forms a table of presence (1) and absence (0) of mammal families across all regions (*i*) for any model (*r*) (see Sheet S1 of the Supplemental Data Appendix for the case with *r* = 7).
*div_i,r_*, *i* = 1,…, *r*, *r* = 5,…, 9: the diversity (or richness) of each region (*i*) of the *r*-region model, computed as the count of families found therein, with $div_{i,r}=\sum_{f=1}^{N}d_{f,i,r}$ (see Sheet S1, Supplemental Data Appendix, for the case of *r* = 7).
*cw_f,i,r_*, *f* = 1,…, *N*, *i* =1,…, *r*, *r* = 5,…, 9: the cosmopolitanism weight indicating whether a family (*f*) found in region *i* is endemic to the region or cosmopolitan (i.e., is found in a number of other regions of the *r*-region world). For example, the cosmopolitanism weight of family *f* of region *i* = 1 in a 7-region world is given as ${cw}_{f,i=1,r=7}={(d}_{f,i=1,r=7}+\sum_{i\neq 1}^{7}d_{f,i,r=7})$ if $d_{f,i=1,r=7}=1$, else ${cw}_{f,i=1,r=7}=0$ (see Sheet S2, Supplemental Data Appendix).
*z_i,r_*, *i* = 1,…, *r*, *r* = 5,…, 9: the “zeroth moment” (or “total cosmopolitanism”) of a given region (*i*) with $z_{i,r}=\sum_{f=1}^{N}{cw}_{f,i,r}$ (see Sheet S2, Supplemental Data Appendix, for *r* = 7).
*z*_*i,r*_/*div*_*i,r*_ = the “mean cosmopolitanism” for a region (*i*) (see Sheet S2, Supplemental Data Appendix, for *r* = 7).
*nz_i,r_*, *i* = 1,…, *r*, *r* = 5,…, 9: the “negative zeroth moment,” in other words, the complementary value to the zeroth moment, defined as $\left(div_{i,r}\times r\right)-z_{i,r},$ where the first term represents the maximum “potential” total cosmopolitanism of a region (*i*) (in a model of *r*-region) with diversity *div_i,r_* (see Sheet S2, Supplemental Data Appendix, for the case where *r* = 7).

In all five (5- through 9-region) models, many of the *N* = 140 mammal families in these extend spatially across two or more of their regions. If one totals up the region-by-region familial diversities arrived at, a figure of $t=\sum_{i=1}^{r}{div}_{i,r}=269$ is produced for the seven-region classification (*r* = 7); for the five-region (*r* = 5) classification, the parallel figure is 225; for six regions, 260; for eight regions, 293; and for nine regions, 307. As a matter of straight combinatorics, one may reasonably ask what the most likely distribution of numbers (i.e, 1 through 7) of regional affinities might be in each case, and consider the results in view of the actual totals. 

The distribution breakdown is given by maximizing $S=N!/\prod_{i=1}^{a}g_{i}!$, where *N* is the total number of groups, *a* is the maximum group size, and $g_{i},\;i=1,\ldots ,a$ is the number of groups with a particular size, so that, for example, if *t* = 269, the combinatorially most frequently arrived at combination of groupings is 1 group with seven elements, 2 with six, 4 with five, 9 with four, 18 with three, 35 with two, and 70 with one. (Results computed herein have been programmed in Python). The total number of groups (*N*) is thus 1 + 2 + 4 + 9 + 18 + 35 + 70 = 139. It is interesting that, in the context of this paper, the observed value for *N* is 140 (mammal families), just one off from 139, and that the number of distinct categories into which these values are distributed is seven. For the *t* = 225, 260, 293, and 307 totals, the combinatorially most frequently arrived at distributions of values also require seven categories of groups, but the total number of groups departs further from 140, being, respectively, 115, 135, 151, and 160. Further, it makes no sense to say that in the five- and six-region classifications there can be a condition wherein a family might exist in seven regions—or, for that matter, that in the eight- and nine-region classifications, it is impossible that a family can exist in that many regions. The comparison between breakdowns of combinatorial likelihood for the five different classifications and their actual totals are given in Table 1. These facts may be added to those produced in [1] in defending the seven-region model of mammal faunas over the other four models, both in terms of its greater efficiency of representation, and the possibility of it being viewed in “natural system” terms.

This is not the end of the matter, however. It should be duly noted that the “max-S solutions” listed in Table 1 above actually do not have any implicit spatial interpretation (as do the “f/g” relations in the same table, based on the real-world totals). Instead, they describe outcomes in much the same manner as the recording of a thousand coin flips, in which mere chance will result in many occasions of multiple “heads” or “tails” in a row being observed. Were the t = 269 elements of the “max-S” 7-region solution merely apportioned into the seven regional units identified in Figure 1 randomly—but weighted according to the differing diversities of each region—we would likely obtain moment measurements (mean, standard deviation, etc.) of each “fauna” that were about equal to one another, and thus quite unlike the observed patterns. As such, any interpretation of the overall system as an evolutionary dynamic would be quite impossible (unless it actually were the case that different areal extents, climates, histories, etc., were irrelevant to the process). What it appears we do have is a biological system whose differentiation in a finite existence (the surface of the earth) has taken place as a result both internal (genetic) forces and the interaction of these with various variable and changing physical inertias (climate, continental placements, etc.) in quite an organized fashion. While many individual instances of speciation may be more or less evident as vicariant episodes, larger-scale patterns require more complex biogeographic models, ones which take into account the pattern and number of continental masses (and their attendant climates and topographical conditions), as well as the effects of differing ecological histories and temporary avenues of faunal communication through short-lived unions (such as those of the Bering Straits and Panama Isthmus). These events may be individually unique, but it is still possible that, in sum, they describe a balance of outcomes that might be described in fairly simple mathematical terms.

## 3. Results

Whether one takes the model presented in [1] to reflect a normative, natural process, or instead just a highly efficient statistical representation, the results can be used to exemplify one potential kind of practical application. This is a form of areal representation, first described in the third 1983 paper, which appeared in the Leigh Van Valen-edited journal *Evolutionary Theory*. In that work, Smith [14] (pp. 226–227) wrote how an efficient faunal systemization might

…be used to characterize the fauna of any particular location on the earth’s surface by first giving attention to the total number of regions that each faunal element found at the given location is present in on a worldwide basis. A distribution of relational values (one for each form specific to the location and ranging in possible magnitude from one to a maximum equal to the number of regions in the classification) can be tallied from which conventional descriptive statistics may be extracted… This approach to data representation might be termed ‘second-order’ by analogy to the second-order (nearest-neighbor) analysis of point patterns; the major difference lies in the fact that a distribution of relative ‘presences’ is obtained rather than a distribution of distances. In the ‘second-order’ representations of organic distribution described here, therefore, attention is focused not on the attributes of any one, pair of, or entire set of locations, but instead on partial orderings of system-level distributional attributes.

**Figure 2:**
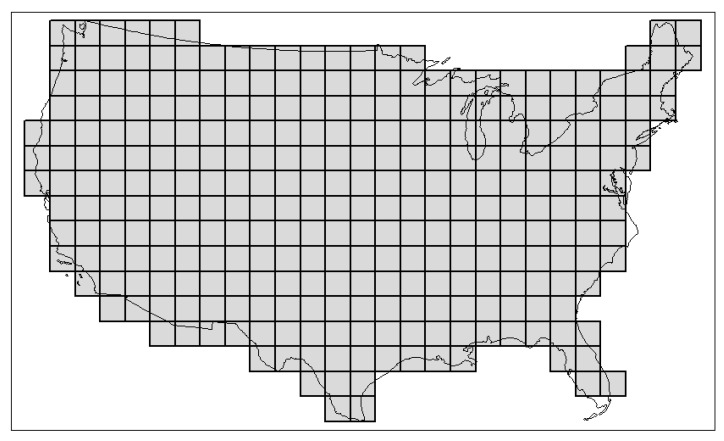
The arbitrarily bounded sample study area investigated here, with a 295-cell grid superimposed.

To illustrate, map figures based on the 1983 mammal region classification [3] were constructed in [14] that highlighted the conceptual appeal of graphically representing “second-order” statistics emerging from the ten-region classification. The mammal families present at 504 worldwide locations were recorded, and the number of the ten regions that each existed in were noted. From such tallies, one could, at each of the 504 locations, calculate a “mean relative cosmopolitanism” statistic that expressed the degree to which each of these point locations is dominated by wide- or limited-ranging forms. (See [14], figure 2). Local values at the high end topped out at 6.5+ regions (in the Arctic), and bottomed out at about 3.5 (in Australia), exposing a domination by generalist families in the former, and by endemic forms in the latter. Another map displayed worldwide “spatial variation in the degree of influence of all specific mammal faunal groupings on the make-up of the faunas at particular locations” ([14], figure 4). Each map relied on the use of isopleths to portray variation in the values obtained.

The examples given in [14] focused on the conditions at a sample of worldwide point locations as they related to the overall world standard. But another advantage of the “second-order” statistics approach is its ability to relate the conditions at any one location not only to the whole, but to (1) any other single location, leading to possible portrayals of tracks and corridors, or (2) the placement and strength of transitions between regions. Some examples follow; these are based on an arbitrarily chosen study area, the conterminous United States. (It should be emphasized that this analysis was constructed for purposes of illustration only, and not as an attempt to augment or supersede the distributional data representations provided by more elaborate studies, e.g., [22,23]).

For purposes of notation, we now assume the present classification model (i.e., Figure 1), wherein the world’s mammal faunal patterns have been arranged into exactly seven geographical regions, *r*; i.e., in this model *r* = 7. In the notation below, an index of *r* = 7 is kept in the statistics to distinguish this specific model from the more general model established in [1] where the *r*-region world is possibly divided into five to nine (or any other number of) geographical areas. Each geographical region in any such seven-region model will be referred to with an index of *i* = 1, 2,… to 7. Mammal families are indexed from *f* = 1 to *N,* where *N* is the total number of mammal families (or “forms”) in the world (140, after [21]), as applied here. 

The statistics defined earlier characterize aspects of a single region (*i*) of the world, and can easily be adapted to a “seven-region world” hypothesis by assuming therein that *r* = 7. Furthermore, statistics pertaining to the analysis of a pair of regions *i,j,* = 1…, 7, can be defined as follows:

*cw*_*f,i,j,r*=7_, *f* = 1,…, *N*, *i,j,* = 1…, 7: the cosmopolitanism weight of a pair of regions indicating whether a family (*f*) found in both regions *i* and *j* is also found in other regions of the seven-region world, with ${cw}_{f,i=1,i=2,r=7}={(d}_{f,i=1,r=7}+d_{f,i=2,r=7}+\sum_{i\neq 1,2}^{7}d_{f,i,r=7})$ if $d_{f,i=1,r=7}=d_{f,i=2,r=7}=1$, else ${cw}_{f,i=1,i=2,r=7}=0$ (see Sheet S3, Supplemental Data Appendix).
*z*_*i,j,r*=7_, *i,j,* = 1,…, 7, the “zeroth moment” (or “total cosmopolitanism”) of a pair of regions (*i,j*). For example, $z_{i=1,i=2,r=7}=\sum_{f=1}^{N}{cw}_{f,i=1,i=2,r=7}$ when $d_{f,i=1,r=7}\mathrm{and}\;d_{f,i=2,r=7}=1$ and 0 otherwise (see Sheet S3, Supplemental Data Appendix).
*nz*_*i,j,r*=7_, *i,j,* = 1,…, 7: the “negative zeroth moment,” of a pair of regions (*i,j*), with $nz_{i,j,r=7}=(c_{i,j,r=7}\times 7)-z_{i,j,r=7}$, where *c*_*i,j,r*=7_ represents the number of common families between two regions, *i* and *j,* in a world of *seven* regions (see Sheets S3 and S4, respectively, for the construction of *nz*_*i,j,r*=7_ and *c*_*i,j,r*=7_). Note that *nz*_*i,j,r*=7_ collapses to $nz_{i,i,r=7}=(c_{i,i,r=7}\times 7)-z_{i,i,r=7}=(div_{i,r=7}\times 7)-z_{i,r=7}$ when *i* = *j*, as the common families between a region (*i*) and itself is indeed the total number of families in that region (i.e., its diversity).
*dis*_*i*,*j*,*r*=7_, *i*,*j* = 1,…, 7: the number of distinct families between two regions, *i* and *j,* in a world of seven regions. In particular, *dis*_*i,j,r*=7_ is the number of families found in *i* but not in *j,* while *dis*_*j*,*i*,*r*=7_ is the number of families found in *j* but not in *i* (see Sheet S5, Supplemental Data Appendix).
*s*_*i*,*j*,*r*=7_, *i* and *j* = 1,…, 7: the Smith similarity index [3] between two regions, *i* and *j*, in a world of seven regions, with *s*_*i,j,r*=7_ = *c*_*i,j,r*=7_ − (*dis*_*i,j,r*=7_ + *dis*_*j,i,r*=7_), or in a short-cut notation, *s*_*i,j,r*=7_ = *a* − (*b* + *c*), where *a* equals the number of families common to the regional pairing in question, and from which we subtract the number of families found in *i* but not in *j* (*b* = *dis*_*i,j,r*=7_), and the number of families found in *j* but not in *i* (*c* =*dis*_*j,i,r*=7_) (see Sheet S5, Supplemental Data Appendix).

In our specific illustration, a regular sample grid of 295 cells was set across an equal-area map of the lower forty-eight (conterminous) states of the United States (Figure 2); the number of mammal families (excluding strictly marine forms) present in each was then tallied, based on an examination of more than a hundred taxonomic-, state-, and regional-level sources. Each cell will be referred to with the index *c* = 1, 2,…, 295. Mammal families in the USA are indexed as *f* = 1,… to *N_US_*, where *N_US_* is the total number of mammal families (32) recorded in the USA. We can now define some statistics for each of these USA sample cells. For convenience and to distinguish this model from the seven-region model above, a *usa* subscript has been added in the notation below. Let us assume the following:

*d_f,c,usa_* for *f* = 1,…, *N_US_*, *c* = 1,…, 295: a dummy variable that takes a value of 1 if there is such a family (*f*) in cell *c*, and 0 otherwise. The collection of *d_f,c,usa_* forms the table of presence (1) and absence (0) of mammal families across all cells in the USA (see Sheet S7, Supplemental Data Appendix).
*div_c,usa_* for *c* = 1,…, 295: the diversity (or richness) computed as the count of mammal families found in each of the 295 geographical cells that make up the USA, with ${div}_{c,usa}=\sum_{f=1}^{N_{US}}d_{f,c,usa}$ (see Sheet S7, Supplemental Data Appendix).
*cw_f,c,usa,r=7_* for *f* = 1,…, *N_US_*, *c* = 1,…, 295: the cosmopolitanism weights of a family (*f*) present in a geographic cell (*c*) of the USA as measured using the count of that family’s presence in all seven regions of the world (not the count of its presence in the 295 cells constituting the USA). Hence, it is computed as ${cw}_{f,c,usa,r=7}=\sum_{i=1}^{7}d_{f,i,r=7}$ if *d_f,c,usa_* = 1_,_ otherwise, ${cw}_{f,c,usa,r=7}=0$, and where *d*_*f,i,*7_ is defined earlier, albeit now restricted to the 32 families (*f*) found in the USA (*f* = 1,…, *N_US_*) (see Sheet S8, Supplemental Data Appendix; Sheet S8 makes itself a reference to Sheet S12, which provides a slice or subset of Sheet S2, keeping only families found in the USA).
*z*_*c,usa,r*=7_ is the total cosmopolitanism statistic (or zeroth moment) of a geographical cell, *c*. It is computed for a *c* as the summation over all families (*f*) found in the USA (*f* = 1,…, *N_US_*) of the cosmopolitanism weights, *cw*_*f,c,usa,r*=__7_, so that $z_{c,usa,r=7}=\sum_{f=1}^{N_{US}}{cw}_{f,c,usa,r=7}=\sum_{f=1}^{N_{US}}(\sum_{i=1}^{7}d_{f,i,7}\;if\;d_{f,c,usa}=1)$. Finally, the mean total cosmopolitanism statistic is *z*_*c,usa,r*=7_/*div_c,usa_* (see Sheet S8, Supplemental Data Appendix).
*s*_*c*1*,c*2*,usa*_: the Smith similarity index [3] between two geographical cells, *c*1 and *c*2, in the USA, with *s_c_*_1*,c*2*,usa*_ = *c_c_*_1*,c*2*,usa*_ − (*dis_c_*_1*,c*2*,usa*_ + *dis_c_*_2*,c*1*,usa*_), where *c_c_*_1*,c*2*,usa*_ is the count of common families across the pair of geographical cells, *dis_c_*_1*,c*2*,usa*_ is the count of families found in *c*_1_ but not in *c*_2_, and *dis_c_*_2,*c*1*,usa*_ is the count of families found in *c*_2_ but not in *c*_1_. In a parallel short-cut notation, we set *s_c_*_1*,c*2*,usa*_ = *a* − (*b* + *c*). Sheet S9 of the Supplemental Data Appendix illustrates the computation of the similarity index between any geographical cell and cell #258 (covering southwestern Georgia).

**Figure 3:**
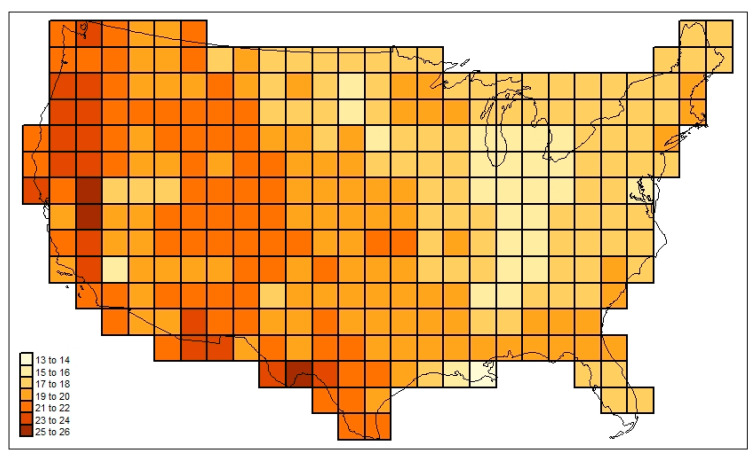
Family-level mammalian richness (*div_c,USA_*) across the sample study area investigated here.

**Figure 4:**
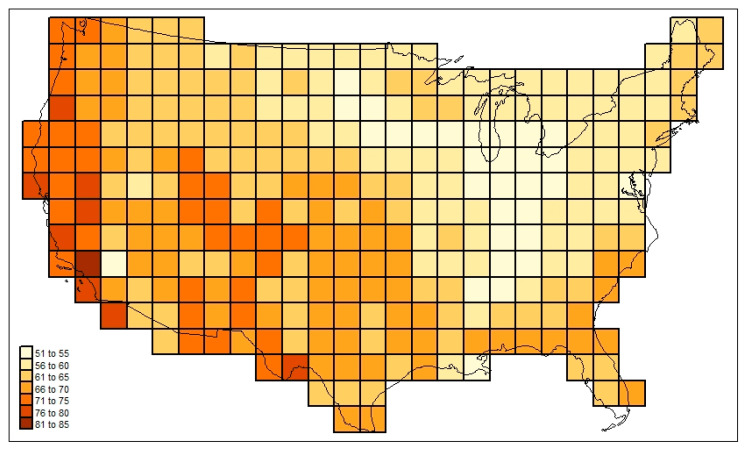
Total mammal family cosmopolitanism statistics (*z*_*c,usa,r*=7_) across the sample study area investigated here.

**Figure 5:**
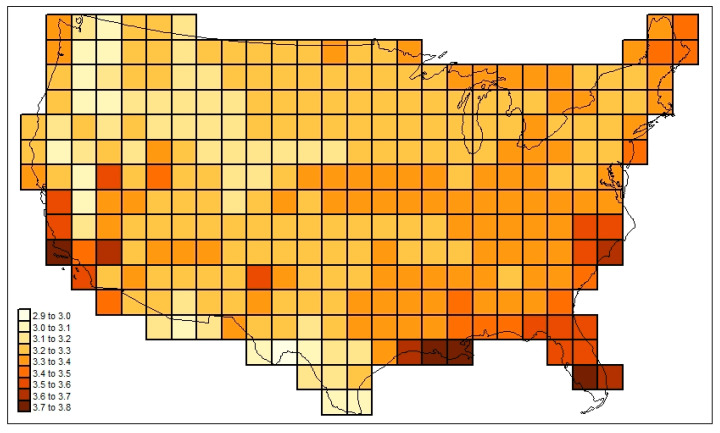
Mean mammal family cosmopolitanism statistics (*z*_*c,usa,r*=__7_/*div_c,USA_*) across the sample study area investigated here.

Figure 3 maps the familial diversities record (*div_c,USA_*) in the 295 cells. Figure 4 maps the associated total cosmopolitanism statistics (*z*_*c,usa,r*=7_), and Figure 5 shows the calculated mean cosmopolitanism values (*z*_*c,usa,r*=7_/*div_c,USA_*). See also Sheet S14 for an R-program that generates all US maps presented in this section.

**Figure 6:**
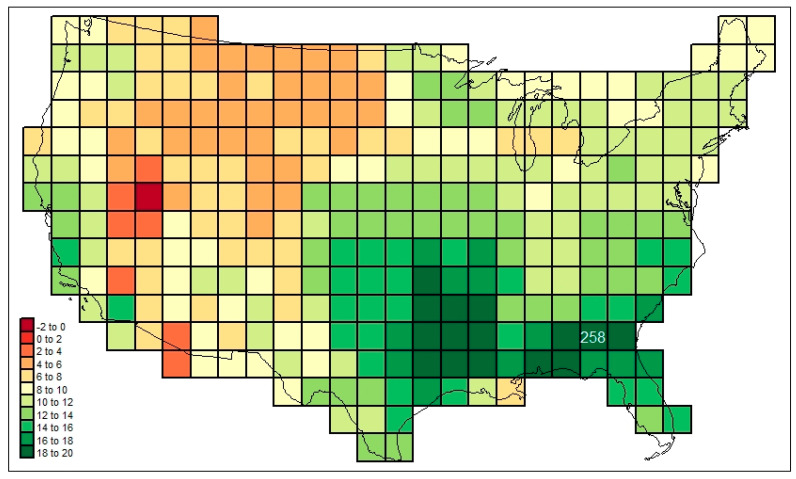
Mammal family similarities with respect to cell #258 (southwestern Georgia) of the grid mapped out in Figure 2.

For our first analytical example, consider how the present model can be used to map the net *similarities* of single cells (or, if desired, point location tallies) in the sample area to any other one. This has been carried out (using the Smith similarity index, a measure of association based on an intersection set approach [3] eschewing proportional conversions) in Figure 6, in which the US-wide mammal family faunal similarities to cell #258 (covering southwestern Georgia) are mapped. (See Sheet S9, Supplemental Data Appendix). In this representation, a “track” of high similarity extends from the area around #258 westward, to eastern Texas and Oklahoma. Dissimilarity maximizes in the Great Basin area. 

**Figure 7:**
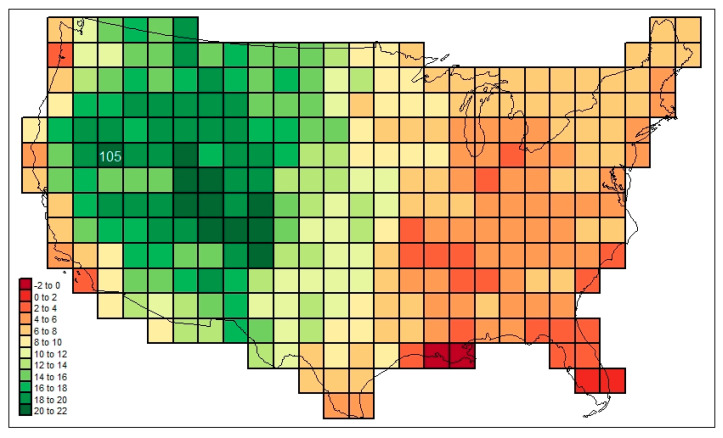
Mammal family similarities in respect of cell #105 (north–central Nevada) of the grid mapped out in Figure 2.

In Figure 7, another such pattern is mapped, this one originating with the mammal families found in cell #105, located in dry north–central Nevada. (See Sheet S10, Supplemental Data Appendix). The falling away of similarities to cell #105 is somewhat more regular here than in Figure 6.

The following are among the things that stand out in Figure 3, Figure 4, Figure 5, Figure 6 and Figure 7: in Figure 3, the low diversities in the eastern Midwest (possibly a function of anthropogenic influence, plus the impact of recent glaciation) and the higher values in areas with a greater elevational/ecological diversity; in Figure 4, lower values in the Midwest and areas of “difficult” ecological conditions (southeastern California, central Nevada, and coastal Louisiana); in Figure 5, lower values in areas of heterogeneous habitats and higher values in dispersal–remote areas (southeastern United States); in Figure 6, the lowest similarities in remote dry areas and a southeastern U. S. corridor effect among high similarities; in Figure 7, a more regular distance–decay effect impacted by the nearby presence of coastal Pacific forms. 

**Figure 8:**
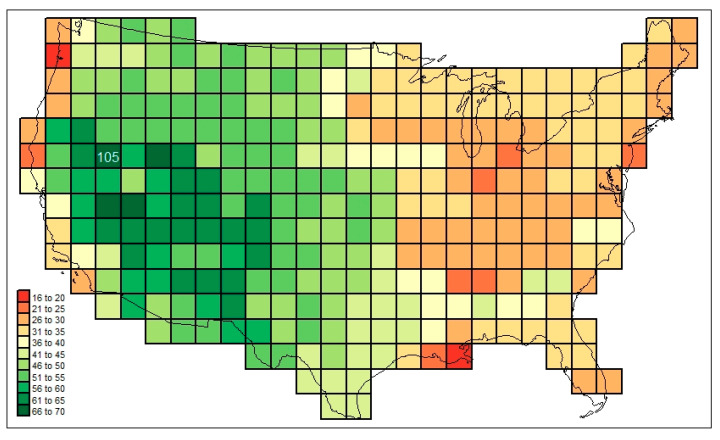
Mammal family similarities in respect of cell #105 (north–central Nevada) of the grid mapped out in Figure 2, weighted based on cosmopolitanism values.

An elaboration on the relational values mapped in Figure 6 and Figure 7 can be created by substituting cosmopolitanism totals for the basic similarities approach applied in these (i.e., presence is weighted according to the number of regions in which each form is found (*cw*_*f,c,usa,r*=7_)). The cosmopolitanism similarity values connected to the similarities mapped in Figure 7 are mapped in Figure 8. (See Sheet S11, Supplemental Data Analysis).

**Figure 9:**
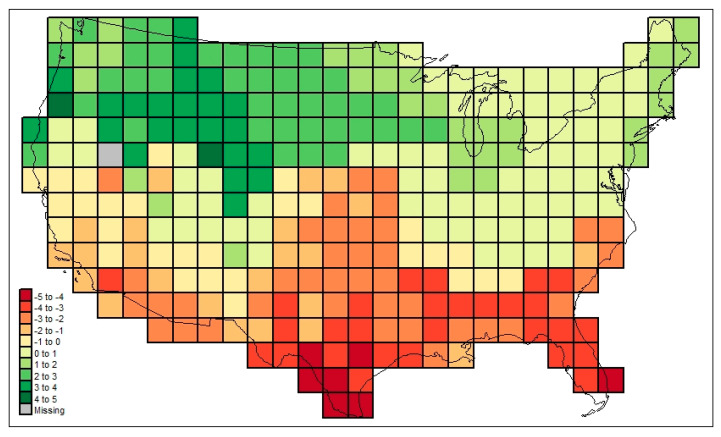
Residuals from a regression of the 294 mammal total faunal cosmopolitanism similarities data of Figure 8 with the simple similarities of Figure 7.

A regression of the Figure 7 values on the Figure 8 ones, as well as yielding a simple Pearson r correlation statistic of 0.9096, produces the set of residuals mapped in Figure 9.

In Figure 9, the residuals from the regression are seen to be highly spatially autocorrelated; that is, the simple regression model may be regarded as invalid as is (violating the standard of the independence of relation between residuals), requiring that additional explanatory variables be brought to bear. In other words, the general distance–decay function of similarity between cell #105 and other cells is secondarily complex: it extends beyond simple considerations of relative distance. Apparently, various historical and ecological forces, as reflected by spatially nonrandom assemblages of families of varying levels of cosmopolitanism, have slowed or accelerated the rates of distance decay. Such forces might be suggested, and examined in turn.

**Figure 10:**
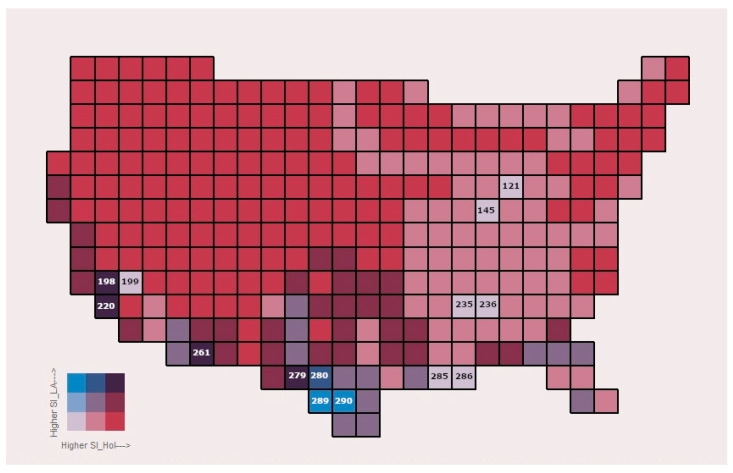
Mammal family faunal similarities (using the Smith similarity index) between individual cells of the sample study area and the overall regional mammal family tallies of the Holarctic and Latin American regions, employing a bivariate mapping technique. Numbers appearing in the figure represent the i.d. number of that cell.

Another possible application of place-specific similarity scores concerns the earlier-alluded-to characterization of transition zones between regions. Treatments of this subject have produced a considerable literature of late (e.g., Marshall and Liebherr [24]; Morrone [25,26,27,28]; Halffter and Morrone [29]; Hermogenes de Mendonça and Ebach [30]; López-García and Morrone [31]). In the present context, evaluation can proceed through comparisons of the faunas at a number of particular peripheral locations with the total diversities assigned to the (usually two, but occasionally three) adjoining regions. This will both isolate the point of transition of primary affinity, and detail its sharpness. This is well illustrated in Figure 10, which maps part of the transition between the Holarctic and Latin American regions using the present example. In particular, for each of the 295 US geographical cells in Figure 10, we compute two Smith similarity indices, one tracking the faunal similarity of any given individual US cell in respect of the whole Holarctic region, and one tracking the faunal similarity of that same US cell in respect of the whole Latin America region. (See Sheet S13, Supplemental Data Appendix). These two indices can then each be discretized in three classes of similarity values (low, medium, high). Using basic principles of bivariate choropleth mapping, we obtain (3)^2^ = 9 classes, each represented by a single color. A cell colored in a mix of dark “blue/red” indicates a high score for both similarity indices, while a combination of pale or light “blue/red” reflects a low score for both similarity indices. This approach allows us to track the transition between the Holarctic and the Latin American regions within the USA. 

Thus, Figure 10 groups the data into pairings of three classes of values combining low/medium/high similarity to both the Holarctic and Latin American standards. The study area here falls nearly entirely within the Holarctic region, so it will not be surprising to find that only three cells (nos. 280, 289, and 290) exhibit higher similarities to the Latin American region than to the Holarctic. These three cells are, as it turns out, right where the Holt et al. data place the northernmost extension of the Latin American region: southern Texas along the Rio Grande (see Figure 1). (Such a placement also provides a crucial validation of the appropriateness of the Smith similarity index here.) Cell nos. 198, 220, 261, and 279 (covering, respectively, expanses north and south of Los Angeles, cross-border southeasternmost Arizona, and the transnational Big Bend vicinity) are areas of relatively high family richness due largely to high environmental diversity, and demonstrate relatively high similarities to both the Holarctic and the Latin American regions. The six lightest-colored cells (121, northwestern Ohio; 145, central Indiana; 199, central Mohave Desert; 235, northern Mississippi; 236, northwestern Alabama; and 285 and 286, bayou Louisiana) cover areas of relatively low topographic/environmental diversity and family richness, leading to low absolute similarity to both the Holarctic and Latin American regions, taken as wholes. The large expanse of pink cells (medium similarity to the Holarctic, low similarity to the Latin American) in the Eastern United States is indicative of low family richness and total cosmopolitanism there, and somewhat elevated mean cosmopolitanism (see Figure 6, Figure 7 and Figure 8); these conditions probably result from relative remoteness and low environmental/topographic diversity, the lagging effects of glaciation, and anthropogenic factors.

One can imagine various ways of extending the transition zone analysis, for example, by varying bin definitions and limits, changing cell tallies by including strictly marine forms, moving to another taxonomic level (e.g., genera), or adding in mean cosmopolitanism statistics to aid in the depictions of the regional standard comparisons.

## 4. Discussion

The thoughts produced here and in [1] are meant to stimulate thinking as to whether a generally stochastic process might, under natural and/or probabilistic constraints, follow through to produce highly idiographic systems whose “program” of development yet remains internally generalizable. We are able to agree that an evolutionary sequence of events likely underlies what we witness about us in the biosphere, but classical Darwinian thoughts on the “random walk” nature of biological evolution have clouded our consideration of the range of ways a generally “stochastic” process might operate. The engineer Adrian Bejan [32,33] has suggested that natural systems evolve by better facilitating the flows of vital nutrients that course through them, but his model does not specify any general principles that might be driving such a process. The present one does, by postulating a model of subsystem (in this case, regional faunas) evolution based on simple “most-probable-state” origination and interaction principles. Apart from analogous empirical investigations (for example, on the manner of originations/interactions among the main tectonic plates or organal systems in the body), it seems that a simulation approach might prove fruitful in identifying unforeseen principles of faunal differentiation, including ones relatable to the kinds of combinatorics discussed here. 

## 5. Conclusions

The present study, an extension of the findings of [1], is intended to provoke discussion as to whether complex natural systems may evolve as the result of a stochastic process internally guided by simple combinatorial organization principles that have not yet been recognized. The results in [1] are, in and of themselves, sufficient to identify a seven-region-based mammal fauna arrangement as being most representative of the distribution patterns in question, but the findings presented here lend more credence to the possibility that something more than just a “random-walk” kind of chance underlies this outcome. That this is more than just a philosophical or theoretical notion is shown by its practical application to cartographic depictions of distribution patterns that are both descriptive in a novel manner and capable of lending their way toward more explanatory models of presence–absence.

## Figures and Tables

**Table 1:** Combinatoric breakdowns for the r totals for the five faunal classifications. Under the “max-S solution” listings, “1/7”, for example, may be read as “one group with seven elements”.

Model	with t=	Max-S Solutions	Total Number of Groups	Actual No. of Mammal Families Present; “f/g” May Be Read as “f Families Present in g Regions”	N, Actual Families
5-region model	225	1/7 2/6 3/5 7/4 15/3 30/2 58/1	116	2/5 5/4 13/3 36/2 84/1	140
6-region model	260	1/7 2/6 4/5 8/4 17/3 35/2 68/1	135	6/5 12/4 11/3 38/2 73/1	140
7-region model	269	1/7 2/6 4/5 9/4 18/3 35/2 70/1	139	3/6 6/5 10/4 12/3 36/2 73/1	140
8-region model	293	1/7 2/6 5/5 10/4 19/3 38/2 76/1	151	3/7 3/6 9/5 6/4 17/3 32/2 70/1	140
9-region model	307	1/7 2/6 5/5 10/4 20/3 41/2 81/1	160	3/8 0/7 6/6 9/5 7/4 13/3 33/2 69/1	140

## Data Availability

Data available in the Supplementary Materials.

## Supplementary Materials

The following supporting information can be downloaded at https://www.mdpi.com/article/10.3390/life13112193/s1.
